# Diversity-aware Population Models: Quantifying Associations between Socio-Spatial Factors and Cognitive Development in the ABCD Cohort

**DOI:** 10.21203/rs.3.rs-4751673/v1

**Published:** 2024-07-30

**Authors:** Nicole Osayande, Justin Marotta, Shambhavi Aggarwal, Jakub Kopal, Avram Holmes, Sarah W. Yip, Danilo Bzdok

**Affiliations:** 1 McConnell Brain Imaging Centre, Montreal Neurological Institute (MNI), McGill University, Montreal, Quebec, Canada.; 2 Mila - Quebec Artificial Intelligence Institute, Montreal, Quebec, Canada.; 3 Department of Psychiatry, Brain Health Institute, Rutgers University, Piscataway, NJ, USA; 4 Department of Psychiatry, Yale University School of Medicine, New Haven, CT, USA; 5 Child Study Center, Yale University School of Medicine, New Haven, CT, USA; 6 Department of Biomedical Engineering, Faculty of Medicine, McGill University, Montreal, QC, Canada.; 7 School of Computer Science, McGill University, Montreal, QC, Canada.

## Abstract

Population-level analyses are inherently complex due to a myriad of latent confounding effects that underlie the interdisciplinary topics of research interest. Despite the mounting demand for generative population models, the limited generalizability to underrepresented groups hinders their widespread adoption in downstream applications. Interpretability and reliability are essential for clinicians and policymakers, while accuracy and precision are prioritized from an engineering standpoint. Thus, in domains such as population neuroscience, the challenge lies in determining a suitable approach to model population data effectively. Notably, the traditional strata-agnostic nature of existing methods in this field reveals a pertinent gap in quantitative techniques that directly capture major sources of population stratification. The emergence of population-scale cohorts, like the Adolescent Brain Cognitive Development^SM^ (ABCD) Study, provides unparalleled opportunities to explore and characterize neurobehavioral and sociodemographic relationships comprehensively. We propose diversity-aware population modeling, a framework poised to standardize systematic incorporation of diverse attributes, structured with respect to intrinsic population stratification to obtain holistic insights. Here, we leverage Bayesian multilevel regression and poststratification, to elucidate inter-individual differences in the relationships between socioeconomic status (SES) and cognitive development. We constructed 14 varying-intercepts and varying-slopes models to investigate 3 cognitive phenotypes and 5 sociodemographic variables (SDV), across 17 US states and 5 race subgroups. SDVs exhibited systemic socio-spatial effects that served as fundamental drivers of variation in cognitive outcomes. Low SES was disproportionately associated with cognitive development among Black and Hispanic children, while high SES was a robust predictor of cognitive development only among White and Asian children, consistent with the minorities’ diminished returns (MDRs) theory. Notably, adversity-susceptible subgroups demonstrated an expressive association with fluid cognition compared to crystallized cognition. Poststratification proved effective in correcting group attribution biases, particularly in Pennsylvania, highlighting sampling discrepancies in US states with the highest percentage of marginalized participants in the ABCD Study^©^. Our collective analyses underscore the inextricable link between race and geographic location within the US. We emphasize the importance of diversity-aware population models that consider the intersectional composition of society to derive precise and interpretable insights across applicable domains.

For decades, neuroscience research has centered on dissecting the intricacies of the adult brain, leaving a conspicuous void in understanding preadolescent brain development—a crucial phase essential for cognitive growth and dynamic processes across the lifespan of brain maturation^1^. It is widely accepted that environmental enrichment and deprivation influence cognitive development, though their specific roles remain vaguely defined^2^. Demystifying how lived experiences shape brain-behavior trajectories is inextricably linked with early childhood exposure and socioeconomic sentinels^1,3^. Despite this knowledge, most existing studies treat sociodemographic variables (SDVs) as a monolithic, ignoring their stratified nature and diverse implications. Within this context, an ongoing question in population neuroscience is how differential effects of socioeconomic factors during early childhood might relate to inter-individual differences in human behavior^4^. However, there is a scarcity of comprehensive datasets providing empirical evidence linking sociodemographic factors and neural correlates of behavioral trajectories. Bridging this gap by accounting for major sources of population stratification would facilitate research that offers more generalizable insights across diverse subpopulations and research areas.

The emergence of population-scale cohorts, like the Adolescent Brain Cognitive Development^SM^ (ABCD) Study, offers unparalleled opportunities to explore comprehensive neurobehavioral and sociodemographic relationships. This collaborative data initiative longitudinally follows a cohort of 11,880 children starting at age 9–10, recruited across 17 American states. Meticulous in its sampling design (cf. methods), the ABCD Study^©^ measures a wealth of attributes encompassing child health and development^5^. These include parent self-report metrics, child-reported support systems, and environmental factors. Here, we leverage this richly phenotyped data resource to characterize sociodemographic predictors of cognitive development, answering some of the most pressing questions in population neuroscience, using our unified platform for diversity-aware population modeling.

Within a developmental context, higher socioeconomic status (SES) is associated with greater access to cognitive enrichment i.e., complex environments with diverse learning materials^6^. In contrast, material poverty during early childhood is a risk factor for mental illness later in life^7,8,9,10^. In the US, 1 in 4 children are born into impoverished households, and studies suggest that spending more time in poverty from birth to age 9 is associated with mental illnesses, personality disorders, and adverse cognitive behavior trajectories as individuals grow into adulthood^10^. Furthermore, research indicates that racialized children are more likely to endure prolonged periods of poverty compared to their White counterparts^11,12^. While these findings provide valuable insights into the dynamics between SES, environmental conditions, and behavioral domains, there is an absence of intersectional perspective, accounting for inter-individual differences. Such strata-agnostic studies miss the ever-shifting interplay between cognitive development and inherent population stratification. Consequently, the oversimplification of familial context downplays the role of systemic privileges that permeate sociodemographic sectors, which may, in fact, serve as direct sources of variation in cognitive outcomes^13^. We assert that studying such mutually related factors within a holistic framework may reveal a greater organizational network underlying cognitive trajectories in preadolescence.

The premise of race and geographic location as key discriminative structures underlying sociodemographic disparities is rooted in both critical race and spatial inequality theories. These fields describe how systemic biases manifest in societal structures and spatial organization, shaping unequal distributions of resources and opportunities^14,15^. Against this backdrop, researchers have described the geography of exclusion as the phenomenon by which race, and segregation confer risk of concentrated poverty^14,16^. They show macro patterns at the “place level” of locally concentrated poverty, primarily disenfranchising Black and Hispanic individuals^16^. Evidence also suggests that the relationships between SES and child development may differ by race, yet at the inter-individual level, much is yet to be pieced together about how race, geography, and SES interact to shape child developmental outcomes^17^. As such, there is a need for quantitative methods that can dynamically incorporate contextual information to capture the interplay of intrinsic hierarchical interactions within sociodemographic diversity predictors of interest.

Despite extensive literature outlining the importance of diversity factors, they are often treated secondary to core scientific queries. These attitudinal barriers have long contributed to a passive approach towards establishing systematic mechanisms that are best suited to incorporate intersectional variables for practical prediction tasks. Thus, standardizing quantitative tools tailored for modeling population attributes is a persistent challenge. Due to a myriad of latent confounding effects that work in tandem with SDVs, some of which are not directly measurable, population-level analyses are inherently complex. In population health research, multilevel modeling has been recognized as a practically useful statistical technique for integrating diverse attributes^23^. However, in the classic frequentist regime, this approach can be quite restrictive due to its reliance on fixed point parameter estimates. Unlike Bayesian frameworks, frequentist models do not account for the uncertainty that is intrinsic to real-world settings.

To overcome these limitations, we adopt a survey estimation approach in which we introduce a proof-of-principle for novel diversity-aware models, designed to grasp intersectional ensembles of group identifiers. We leverage a Bayesian multilevel regression and poststratification (BMRP) strategy, to enable accurate and interpretable predictions of population-level data. Varying-intercepts and varying-slopes models are ideal for providing individualized predictions and seamlessly integrating common and individual sources of variance. Coupled with poststratification, a powerful statistical technique that incorporates census auxiliary information to correct model estimates for known differences between a sample and the target population, BMRP assumes a dynamic data generation process that enables granularity^24,25^. We employed poststratification to mitigate potential systematic sampling discrepancies of race, state, and sociodemographic strata. This approach ensures accurate predictions for the target population, minimizing the influence of subgroup representation disparities and group attribution biases in our diversity-aware population models. Here, we determine how a constellation of sociodemographic predictors drive variation in cognitive ) outcomes differentially across race and state subgroups. We utilize 3 behavioral domains as ‘ target phenotypes: mental health, personality, and cognition, outlined by Chen et al.^77^ as having shared and unique brain network features, to determine how their relationships with SDVs are intertwined.

We investigated how cognitive development varies as a function of sociodemographic extremes across race and state strata in the ABCD study^26,27^. Emphasizing BMRP as a prime candidate for interpreting complex population dynamics, we uniquely uncovered behavioral profiles that are generalizable, yet unambiguous to specific pockets of people^28,29^. We demonstrate that BMRP can be tailored to develop diversity-aware population models, providing key insights that prioritize interpretability, reliability, precision, and accuracy.

## Results

### Diversity-aware population models elucidate inter-individual differences in cognitive development

**Figure 1:**
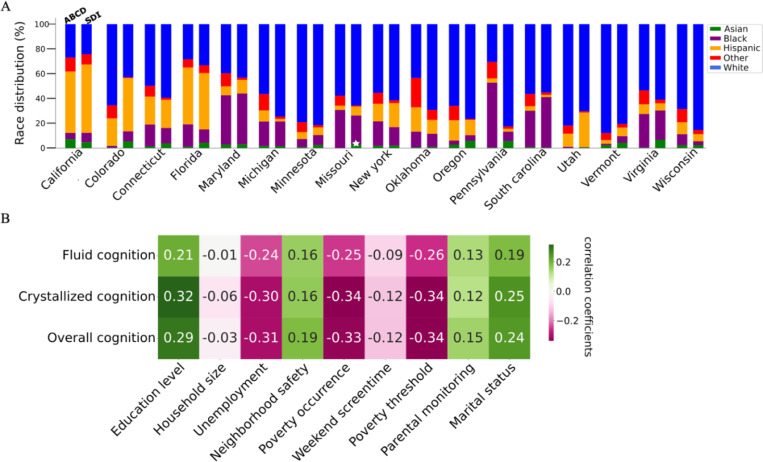
The participant distribution disparities across major sources of population stratification exposes sampling discrepancies in the ABCD cohort. A) A comparison of participant distributions in the ABCD cohort against the US census SDI reveals that race and state strata in the ABCD are not representative of the target population. In the ABCD, Asians were predominantly sampled in California, with sparse representation in other states; namely, there were no Asian participants in Missouri. B) We employed Pearson’s cross correlation to determine the strongest SDV-neurobehavior relationships to model. Based on our set threshold of |p| ≥ 0.2, we identified 14 pairs (out of 2052 candidate pairs) with the strongest positive (green) and negative (pink) associations, narrowing our study to 5 SDVs and 3 cognitive phenotypes. Each pair was then individually modeled with a varying-intercepts and varying-slopes specification to further elucidate how SDVs are differentially associated with cognition across race and state subgroups.

We set out to systematically standardize a proof of principle for diversity-aware population modeling, using Bayesian multilevel regression and poststratification (BMRP) to generate individualized predictions of neurobehavioral phenotypes. However, sampling discrepancies in the ABCD cohort (Fig. 1a) could lead to greater heterogeneity in demographically defined subgroups, potentially impeding the generalizability of these predictions to underrepresented groups. To mitigate this, we used the auxiliary US census social deprivation index (SDI) to show how the poststratification step was poised to reconcile bias and fairness in our model’s estimates. This approach was especially efficient for under-sampled subpopulations and for ensuring true representation of the target population.

In the ABCD cohort, participants’ race is classified into a five-level ethno-racial construct: Hispanic, non-Hispanic Asian, non-Hispanic Black, non-Hispanic White, and Other, derived from the NIH Minimum Reporting guidelines and the Office of Management and Budget (OMB) standards used by the US Census Bureau^89^. While we use this race measure as part of our multilevel modeling structure, we are mindful that these categories are oversimplified and do not fully account for the depth of historical and post-colonial contexts, contributing to the erasure of indigenous peoples and intersectional identities in research.

In the present study, we addressed two central questions: 1) What insights about inter-individual differences in cognitive outcomes can we extract from state- and race-specific sociodemographic variable (SDV) effects? And 2) How are the stratified SDV associations with cognitive development jointly distributed? Our diversity-aware population model offers a unified platform and enables nuanced understanding of the model’s reliability within each demographically defined subgroup, demystifying the complex and intertwined relationships that characterize cognitive phenotypes.

**Figure 2:**
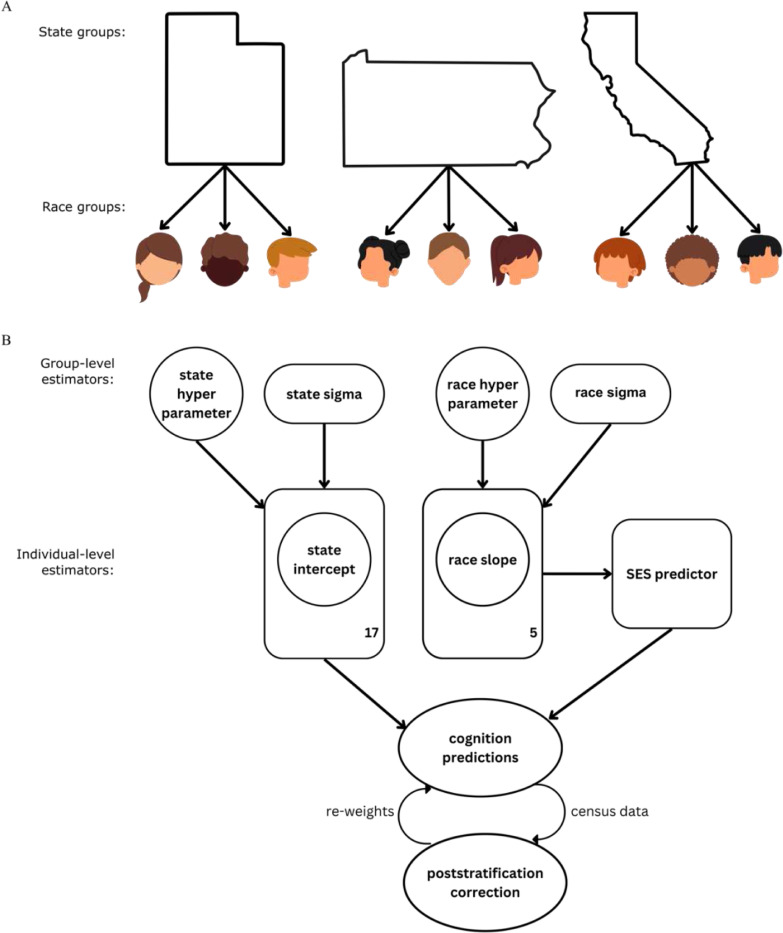
Diversity-aware population modeling schematic. A) The ABCD cohort, partitioned by race and state strata, provides a critical backdrop for our Bayesian multilevel regression and poststratification framework. B) Model specification: We defined the varying-intercepts and varying-slopes model with a state-specific intercept term, estimating 17 baseline cognitive outcomes, and a race-specific slope parameter, estimating 5 sociodemographic effects. The hyperparameters, ergo the state- and race-wide parameters, capture the group-level distribution, which informs the individual-level slope and intercept distributions. The model estimated the relative statistical strength of the state-intercepts in competition with the race-specific SDV effects to predict cognitive development. For the poststratification step, we integrated the auxiliary US census SDI dataset, performed a posterior predictive check, and calculated the data weighted average to correct the estimates. Poised to mitigate subgroup representation biases, we compared the biased predictions against the poststratification results, analyzing for uncertainty intervals, magnitude, and direction.

To carry out our cross-sectional analysis we: 1) aggregated 21 sites into 17 US states (cf. methods) to ensure model stability and geographic compatibility with the US census auxiliary data, 2) curated a shortlist of 36 neurobehavioral phenotypes and 57 SDVs, 3) employed Pearson’s cross correlation (Fig. 1b), to determine the strongest (|p| > 0.2) SDV-neurobehavior relationships to model, identifying 14 pairs (out of 2052 candidate pairs), 4) constructed 14 varying-intercepts and varying slopes (VIVS) models to thoroughly investigate each SDV-neurobehavior pair (Fig. 2a-b), and 5) performed posterior predictive checks to validate our models, then integrated the auxiliary US census SDI into our fitted models for the poststratification step. Given our framework (Fig. 2a-b), we effectively characterized sociodemographic predictors of cognitive development by systematically quantifying socio-spatial effects, thereby revealing inter-individual differences within our target population. Namely, we investigated the stratified effects of 5 SDVs—unemployment, poverty occurrence, poverty threshold, marital status, and educational attainment—and their associations with fluid, crystallized, and overall cognition.

### Neighborhood unemployment exhibits varied associations with cognitive phenotypes across racial groups

**Figure 3.**
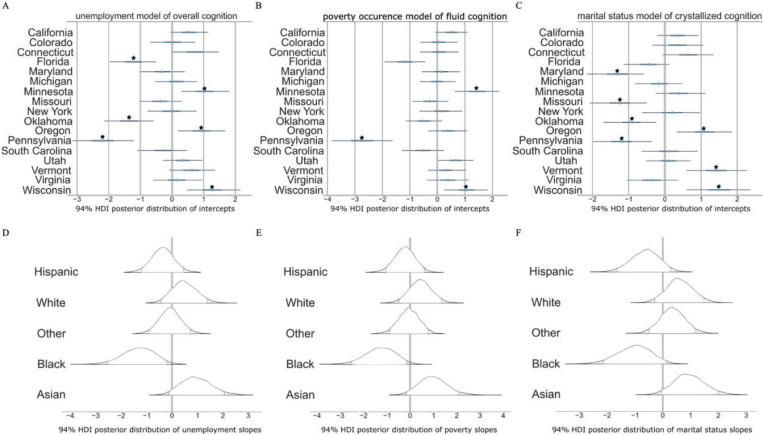
Race-slope effects show wide uncertainty estimates, varying significantly between subgroups. A-C) The posterior distributions of varying-intercepts quantified distinctions in cognition at the state level, with comparable uncertainty intervals, affirming the reliability of the model’s estimates. Pennsylvania stands out as having the strongest negative cognitive outcome, while Wisconsin and Vermont both have strong positive associations. D-F) The varying-slopes show that unemployment, poverty, and marital status exhibit stratified patterns and race-specific disparities in their influence on cognitive development. However, greater uncertainty intervals for Black and Asian SDV effects, conveys less reliability for these subgroups. Thus, SDVs, while robust predictors of cognition, demonstrate non-uniform effects at the race level and partial predictive power.

To investigate individual differences in cognitive phenotypes as a function of neighborhood unemployment, we constructed three VIVS models to predict fluid, crystallized, and overall cognition. The explained variance of overall cognition predictions, as measured by the Bayesian R^2^ score, was 0.37, meaning that unemployment comprised 37% of the variation in this model^30^. The race-slopes exerted greater statistical strength in the model compared to the state-intercepts, making them the primary drivers of variability in the overall cognition predictions. Select states showed strong associations with overall cognition (Fig. 3a), while the posterior distributions of other states hovered near the group mean, displaying non-distinct cognitive outcomes, irrespective of unemployment severity. Upon examination of the direction, magnitude, and uncertainty intervals of each estimated race-specific slope parameter (Fig. 3d), we found that unemployment was not definitively associated with adverse cognitive outcomes for some race subgroups. Namely, for the Asian subgroup, the 94% posterior density interval (HDI) of slopes ranged from −0.2 to 1.9, with a mean of 0.98. Given that we z-scored the target phenotype, this indicated that neighborhood unemployment among Asian children was not as negatively linked to cognition comparative to the race-wide group mean outcome.

Further, unemployment was a robust predictor of crystallized cognition, aligning consistently with overall cognition patterns observed across population strata. Crystallized cognition, defined by the application of acquired knowledge and experience, exhibited significant variability between the state intercepts, primarily characterized by low-employment neighborhoods. In neighborhoods with higher employment rates, crystallized cognition outcomes were virtually indistinguishable between race and state subgroups. This finding confirmed that lower unemployment rates were the driving source of variation in association with crystallized cognition.

Our model of fluid cognition, described by problem-solving abilities and adaptability to new situations, portrayed varying associations with unemployment across race subgroups. The posterior distribution of slope effects for the Hispanic and Other subgroups were closely mirrored, with an HDI of −1.0 to 0.7, which was consistent with estimated parameters in the overall cognition model (Fig 3d). This range represented both the narrowest uncertainty intervals and the smallest deviations from the group-wide unemployment effect. Thus, our findings suggest a generalizable relationship between fluid cognition and unemployment in these race subgroups. Additionally, we explored the transregional association of unemployment effects, facilitating a comparison of race-specific slopes across states. The evidence highlighted variations in the impact of unemployment at the state level, where each race group exhibited either improved or diminished cognition outcomes within specific states. Notably, in Wisconsin, the effects of unemployment in the Black and Hispanic subgroups were more positively skewed than in Pennsylvania and Maryland. Our results demonstrated the joint probabilities of race and state parameters, revealing a regional influence on the direction and magnitude of race-slope unemployment effects on fluid cognition.

The state-intercepts for Florida, Oklahoma, Maryland, and Pennsylvania had a dominant influence in our models of unemployment, across all cognitive domains. As a result, in low-employment neighborhoods, the posterior predictions of cognition were below the state-wide average for all race groups in the aforementioned states. These results may allude to potential disparities in state-level resources and interventions, underscoring a systemic interplay that can amplify the adverse implications of unemployment on cognitive trajectories for children in low SES households.

For the poststratification step, we employed the US census social deprivation index (SDI) dataset (160,436,625 households), open-sourced by the Robert Graham Center (RGC)^74^. In each fitted model, a poststratification cell, *c*, constituted one of 170 possible categories, delineated by the unique combination of 17 states, 5 races, and the binary SDV predictor. To rectify subgroup representation bias, poststratification adjusted each cell’s estimate to accurately reflect the true population composition of each category. We then compared the original and de-biased posterior predictive distributions of cognition scores to determine how the subgroup-representative sample differed at the race- and state-level.

**Figure 4.**
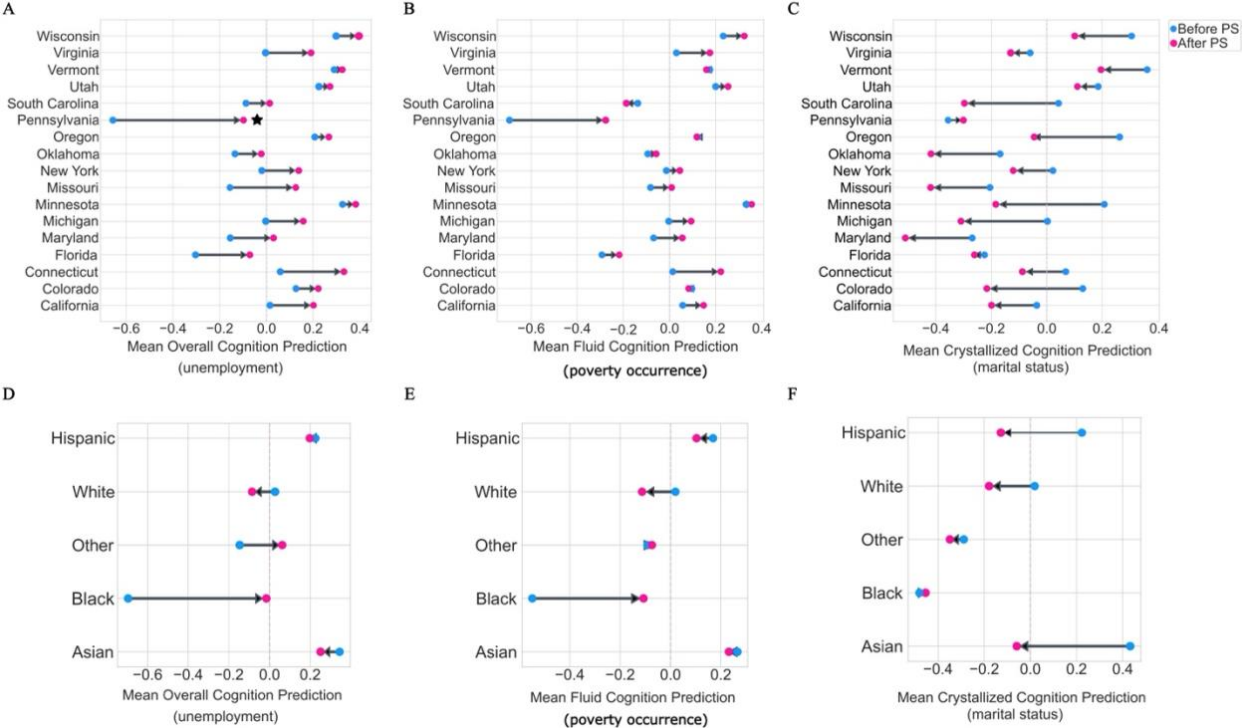
Poststratification mitigates representation bias in state and race subgroups. The integration of US census SDI data enhances the robustness of our diversity-aware population model, effectively de-biasing the predictions. A-C) The posterior predictive distribution is compared against the poststratified posterior predictions of overall, fluid, and crystallized cognition, summarized across states. D-F) Poststratification diminishes the model’s susceptibility to biased predictions across race subgroups, indicating that race SDV effects can be modeled hierarchically without detrimental consequences to the efficacy of the framework.

**Figure 5.**
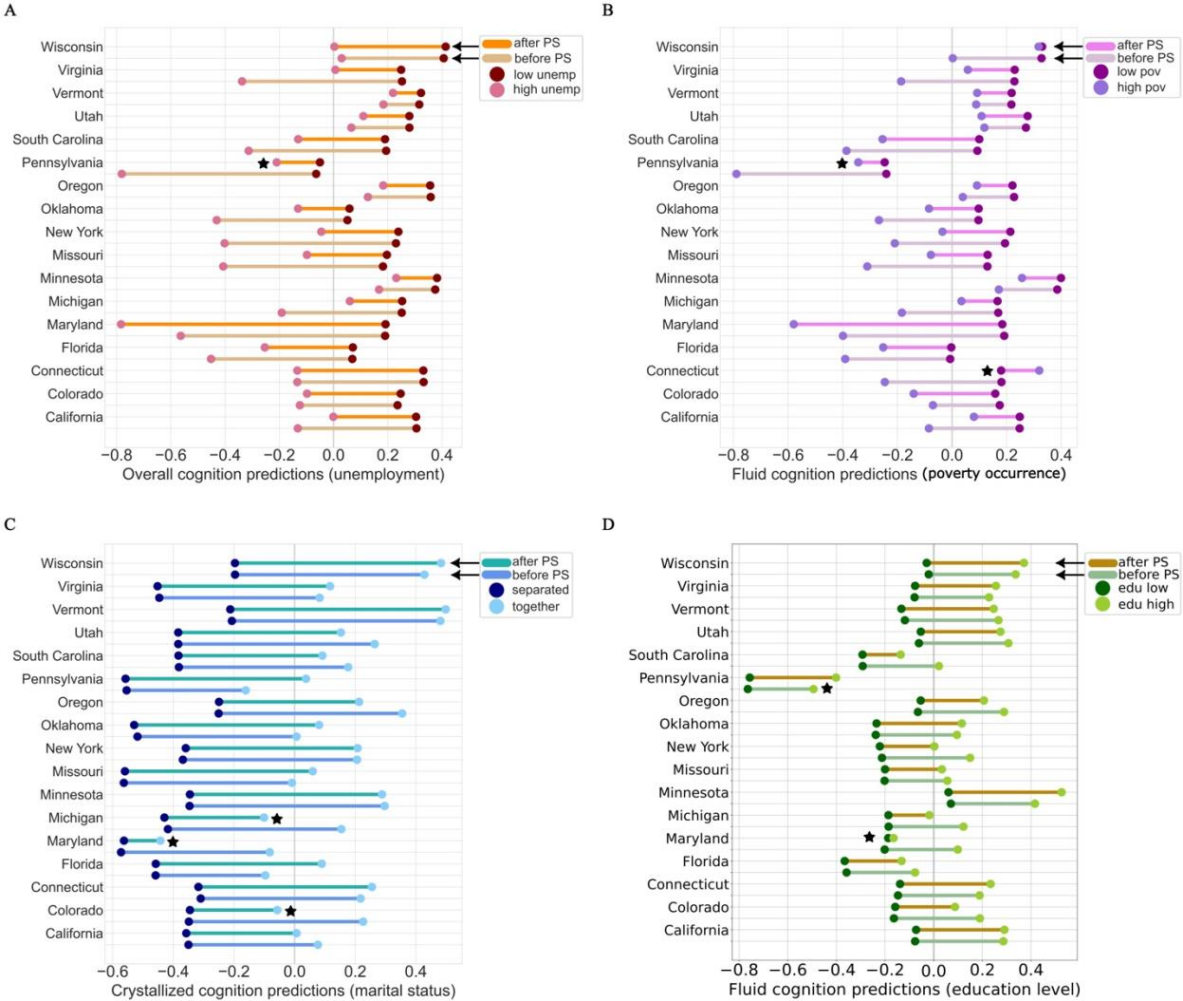
In-state sociodemographic-level disparities are a determining factor of cognitive development profiles. We examined whether cognitive predictions could be distinguished based on state-SDV strata by aggregating posterior predictive samples across 17 US states. Evidently, high educational attainment and two-parent households were associated with robust positive cognitive outcomes, whereas unemployment and poverty had adverse effects. The conditional effects of each SDV predictor exposed certain states with a propensity for diminished cognitive development or heightened vulnerability to lower socioeconomic standings. Pennsylvania exhibited the most pronounced negative deviation from the group mean, while Maryland showed the largest absolute difference in cognitive outcomes between SDV-levels. These findings suggest that Maryland and Pennsylvania were disproportionately impacted by factors such as poverty, unemployment, and single parenthood, whereas in states like Wisconsin, Vermont and Utah, disparities in cognition were more strongly associated with high socioeconomic status, and less sensitive to low socioeconomic standing.

We refined our unemployment model’s posterior predictions of cognition, using the auxiliary US census SDI nonemployed variable. This represents the percentage of individuals between 16 and 64 years of age that are unemployed and not actively seeking work. In Pennsylvania, the initial mean overall cognition prediction of −0.66 was adjusted to a de-biased mean prediction of −0.1 (Fig. 4a). This suggested that unemployment was, in fact, not a distinguishable factor of childhood cognitive development in Pennsylvania (Fig. 5a), revealing misalignment in the real-world representation and the ABCD cohort (Fig. 7). Evidently, the mean crystallized cognition prediction shifted from −0.39 to 0.16, highlighting a change in the direction of the magnitude after poststratification. In California, accounting for 3 of the 21 sampled sites in the ABCD, additional discrepancies surfaced for both crystallized and fluid cognition, after the poststratification step. Areas with low employment rates were counterintuitively associated with above-average cognitive performance. Across the remaining states, differences in cognitive outcomes between low and high employment neighborhoods were effectively dampened. However, in Maryland, we observed a more pronounced cognitive decline associated with low employment. These findings underscore the importance of poststratification in ensuring that our estimates for demographically defined subgroups are reliable and interpretable.

### Poverty effects associated with differentiable cognitive outcomes undergo major poststratification corrections

Our next objective was to quantify the predictive relationships of two poverty indicators—one measuring the occurrence of poverty and the other gauging neighborhoods below the federal poverty threshold—for each of the same three target phenotypes: crystallized, fluid, and overall cognition. We constructed a total of six separate VIVS models that each considered both state-specific distributions of cognition scores and the influence of living in poverty across race subgroups. The models, each achieving a Bayesian R^2^ score of 0.37, exposed distinct state- and race-level differences (Fig. 3b,e). Poverty occurrence was associated with a more pronounced negative effect on cognition for Black children, than any other race group in similar economic conditions (Fig. 3e). We observed that the associated effect of poverty on cognition for Other and Hispanic subgroups was slightly discernible but mostly centered around the group mean. This indicated that neither poverty occurrence nor poverty threshold were strong predictors of their cognition outcomes.

The posterior predictions of fluid cognition scores uncovered distinguishable state profiles when juxtaposing high-poverty with low-poverty neighborhoods. In neighborhoods with low poverty occurrence, the mean predicted fluid cognition score for each state, ranged from 0.1 to 0.4, except for Pennsylvania and Florida (Fig. 5b). Vermont exhibited less disparate fluid cognition outcomes between high- and low-poverty occurrence, suggesting a relatively homogeneous association between poverty and fluid cognition across race subgroups, and a more pertinent region-specific variation. Conversely, Florida, Maryland, South Carolina, and Pennsylvania were characterized by lower fluid cognition outcomes under conditions of high-poverty occurrence, with mean predicted scores of −0.38, −0.40, −0.38, and −0.8, respectively. These states demonstrated a steeper poverty-level disparity in cognition, reflecting a larger population of race groups that were disproportionately affected by poverty occurrence. In Pennsylvania, the mean predicted fluid cognition score for children living in neighborhoods below the federal poverty threshold aligned with the group average. This suggested that poverty threshold had a stronger association with crystallized and overall cognition compared to fluid cognition in Pennsylvania.

We then integrated the SDI federal poverty level (FPL) variable, as a basis to poststratify our posterior predictions of cognition in each of the six models (cf. above). Across the poverty occurrence models, Colorado, Minnesota, Oklahoma, Oregon, Utah, and Vermont, were among the states with the least discrepancies after poststratification (Fig. 4b). Before de-biasing, our results indicated that the prevalence of poverty was linked with stronger negative fluid cognition outcomes across all states. Yet, in our de-biased models, children in Connecticut exhibited a higher mean corrected score of 0.32 in neighborhoods with high-poverty occurrence, versus 0.18 in areas with low poverty occurrence (Fig. 5b). This trend was not observed in any other state, showcasing a unique region-specific effect in Connecticut.

Among the poverty threshold models, the poststratified crystallized cognition predictions stood out, showing that both Maryland and Utah deviated from the state-wide mean outcome. In the original model, living in neighborhoods below the federal poverty threshold did not have a strong association with crystallized cognition in Utah. However, the de-biased results suggested that Utah residents living below the poverty threshold had the lowest predicted cognition score after Maryland. Further, the de-biased crystallized cognition predictions suggested that Pennsylvania was not as negatively impacted by poverty threshold effects as was deemed in the original model. Thus, without poststratification, individualized predictions, particularly from sparsely sampled strata, could have been misleading due to group attribution biases, potentially impeding the efficacy of the model’s predictions.

Our poststratification results unveiled key distinctions between our poverty occurrence and poverty threshold models, despite using the same SDI FPL variable. After de-biasing, the fluid cognition predictions for children living in neighborhoods above the poverty threshold were non-differentiable between race subgroups but *were* differentiable for those living in neighborhoods with low poverty occurrence, showing more varied associations between race subgroups. Interestingly, the most positively skewed de-biased predictions were for Asians living below the poverty threshold. Yet, in terms of poverty occurrence, the most negatively skewed de-biased predictions were for Asians living in relatively well-off neighborhoods. These results may have stemmed from differences in the definitions of the ABCD poverty occurrence variable and the SDI FPL variable, or from variations in how subgroups were affected by poverty predictors.

### Associations linking marital status with cognitive phenotypes are contextually dependent on race and state subgroups

**Figure 6.**
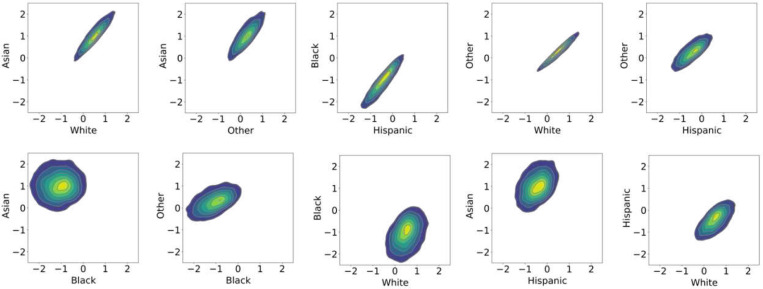
Race slope parameters show overlapping, high-confidence effects of two-parent households on overall cognition. Kernel density estimates convey the varying influence of marital status (SDV) on overall cognition. Notably, we observe a strong, consistent, and predictable association between two-parent households and overall cognition outcomes in Black and Hispanic groups, represented by tightly concentrated, linear-shaped contours. This observation signifies that the direction and strength of this relationship can be reasonably anticipated, given the uniformity within their joint slope distributions, reflecting a dependable impact of marital status within these groups. In contrast, the wider spreads, and circular-like shapes within the joint distributions of the Hispanic and Asian groups denote a more diverse spectrum of outcomes. This indicates that the effect of two-parent households on cognition does not exhibit the same consistency between Hispanics and Asians. These findings accentuate the efficacy of our BMRP strategy in revealing the multifaceted nuances within diverse demographic subpopulations.

**Figure 7:**
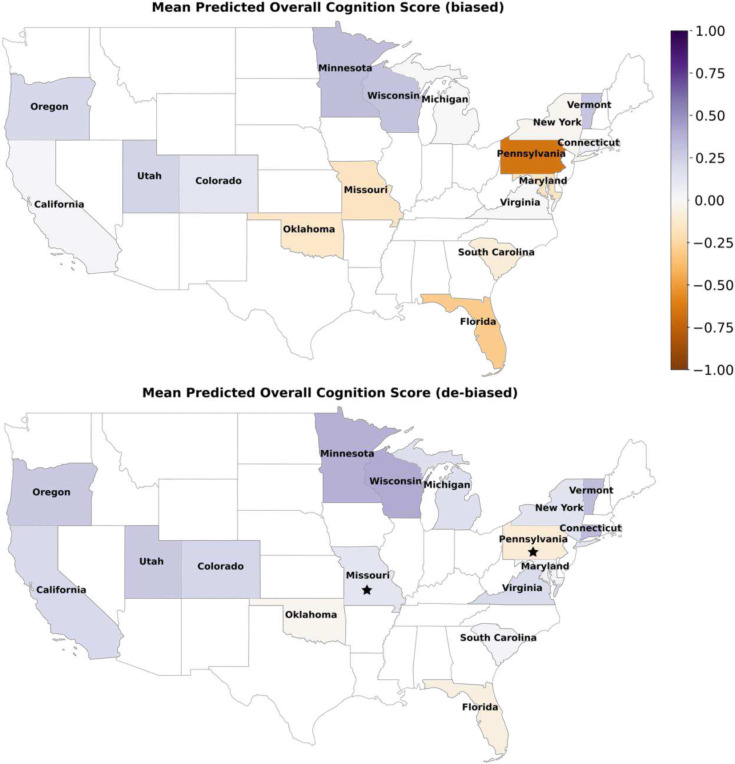
Poststratification successfully mitigates group attribution bias, correcting overall cognition predictions based on unemployment in Pennsylvania. We incorporated US census SDI data into our diversity-aware population models, broadening the basis for parameter estimates and posterior predictions to ensure that our models are reliable and representative of the target population. Given the known population proportions of race-state-SDV strata in the auxiliary SDI, we employed an average weighted sum of posterior predictive samples to adjust for subgroup representation bias in our models. (top) In our original (biased) model of overall cognition as a function of unemployment, Pennsylvania had the lowest mean predicted outcome, while Minnesota, Wisconsin, and Vermont had the highest. (bottom) The de-biased mean predicted overall cognition scores shows that Pennsylvania and Missouri exhibited the strongest poststratification results. This highlights variations in sampling quality across states, where well-sampled states remain stable even after incorporating auxiliary information.

We established two models to explore the associated impact of marital status on crystallized and overall cognition. Across race subgroups, single-parent households were linked to proportionate effects on crystallized cognition, with a mean predicted score of −0.4. The most notable variation occurred in two-parent settings, where the mean predicted crystallized cognition score was higher, particularly for Asian and White children (Fig. 3f). However, among Black children, this difference was marginal, being lower in a two-parent dynamic, indicating a weaker association between marital status and crystallized cognition. To probe our overall cognition model for its predictive reliability between the multilevel parameters, we performed a joint distribution analysis of the race-slopes. This revealed reliable effects of dual parenthood in Asian:White, Asian:Other, Black:Hispanic, and Other:White subgroups, with similar patterns emerging between these parameter pairs (Fig. 6, top). In contrast, the joint distributions of Asian:Black, Black:Other, Black:White, and Hispanic:Asian slope parameters depicted greater dispersion (Fig. 6, bottom), indicating that the effect of two-parent households on overall cognition was not comparable between these subgroups.

We utilized the SDI single-parent household attribute for poststratification, a score quantifying the percentage of single-parent families with dependents aged 18 years or younger. For crystallized and overall cognition, Minnesota exhibited the most significant corrections, with a biased mean prediction of 0.21 and 0.33, and a de-biased mean prediction of −0.19 and −0.04, respectively (Fig. 4c). The mean predicted crystallized cognition score was higher in two-parent households across all states (Fig. 5c). However, the range of predictions were negatively skewed, with the highest mean predicted outcomes observed in Vermont and Wisconsin at 0.45, compared to the most negative scores of −0.58, −0.57, −0.56 in Maryland, Pennsylvania, and Missouri, respectively (Fig. 5c). The mean predicted crystallized cognition score for children with single parents remained consistent after poststratification, across states. However, for two-parent families, the mean crystallized cognition score after de-biasing was slightly more varied. In Maryland, Colorado, and Michigan, the mean predicted crystallized cognition score for children in two-parent households was lower after poststratification. In contrast, in Florida and Pennsylvania, the mean predicted crystallized cognition score for children in two-parent households was higher, after poststratification (Fig. 5c). The de-biased predictions in Maryland were similar in single- and two-parent households, suggesting that in this state, marital status was not a strong predictor of crystallized cognition. As a whole, cognition scores after poststratification were generally predicted to be lower, suggesting that the biased model may have overestimated the relationship between marital status and cognition (Fig. 4c). Thus, poststratification helped us hone the predictions for precise and accurate small-area estimations.

### Educational attainment demonstrates an organized pattern of predictive influence on cognition

Educational attainment represents the proportion of individuals within a neighborhood who have achieved at least a high school education or higher. We constructed three models to investigate how residing in an area with a relatively higher level of education relates with fluid, crystallized, and overall cognition. The state intercepts demonstrated tighter uncertainty intervals of fluid and overall cognition scores, except for Pennsylvania, where the distribution of probable intercept values was wider and negatively skewed. Similar patterns emerged in our crystallized cognition model, albeit with a less variable uncertainty range, fluctuating from −2 to +2, compared to other models ranging from −4 to +2. A consistent race-slope estimation across the three cognitive phenotypes suggested that education had an organized pattern of predictive influence on cognitive development. Higher neighborhood educational attainment was positively linked with cognitive outcomes for White and Asian preadolescents. Otherwise, educational attainment showed neutral associations with cognition. In our overall and fluid cognition models, the mean predicted outcome in both lower-educated and higher-educated neighborhoods was significantly lower in Pennsylvania compared to other states (Fig. 5d). However, the difference between cognition scores in lower-educated and higher-educated neighborhoods was consistent across all states, suggesting that there were regional-driven stratification effects at play (Fig. 5d).

For the poststratification step, we used the SDI education variable, representing the percentage of the US census population aged 25 years or older, with less than 12 years of education. In our crystallized and overall cognition models, Connecticut saw the most drastic change, with a biased mean prediction of 0.11 and 0.07, and a de-biased mean prediction of 0.40 and 0.34, respectively. In our de-biased fluid cognition model, the mean predicted outcome for the higher-educated neighborhoods of Maryland, was lower in comparison to the original model. This indicated less variability between neighborhoods with low- and high-educational attainment in association with fluid cognition (Fig. 5d). In lower-educated neighborhoods, we observed a similar range of predictions across race subgroups. The de-biased model showed that the association between lower-education and cognition was proportional across race subgroups, with a mean predicted cognition score of −0.3. However, the association of higher-educated neighborhoods with cognition was disproportionate across race subgroups, exhibiting diminished returns among Black and Hispanic preadolescents.

The BMRP framework demonstrated its utility in capturing more complex relationships between SDVs and behavioral phenotypes. By means of partial pooling, the model’s exploration of multilevel SDV predictors and state-specific outcomes revealed a higher organizational network of effects, quantifying how individual and group sources of variance influence childhood cognitive development. Poststratification emerged as a crucial technique for enhancing reliability. While Bayesian multilevel regression alone is advantageous for interpretability, the inclusion of poststratification ensures that these methods are applicable in domain-specific contexts and are generalizable across underrepresented populations. Our analysis highlighted that, without poststratification, population models could be prone to inaccuracies. Consequently, our standardized diversity-aware modeling approach underscores the significance of considering intersectional experiences in generative population models.

## Discussion

In population neuroscience, sociodemographic impacts on cognitive development have traditionally been studied under a strata-agnostic lens that neglects contextual parameters related to a population’s composition. Though many studies have examined the interplay of race, geographic location, and other sociodemographic factors by investigating their interactions, we took a different approach^31,33,35,37^. Here, we defined race and state groups in the ABCD Study^©^ as multilevel strata, demonstrating that the effects of sociodemographic variables (SDVs) exhibit stratified patterns, even though race and state groups were not treated as predictors in our models. Our study extends the work of Park et al., drawing on the modeling approach used for small-area estimation and survey methodology to design domain-agnostic diversity-aware population models^24^. Utilizing the uniquely rich phenotyping of ~10,000 youths from the ABCD cohort, we aimed to systematically characterize major sources of variation in cognitive development at the population level, as a prerequisite to refine predictions at the single subject level. Our collective analyses provide evidence that cognitive predictions exhibit systemic SDV effects, highlight spatially concentrated poverty, and demonstrate the ability of poststratification in achieving reliable and interpretable insights across demographically defined strata. We demonstrate that Bayesian multilevel regression is well suited for capturing partially pooled effects, identifying how the model encodes aspects of skewed sampling bias, and providing a full specification of probabilistic uncertainties.

SDVs and systemic privileges are inherently intertwined by virtue of salient societal axes; together, they show a well-established dependence on a diverse array of behavioral phenotypes^31,32^. The cross-cultural effects of SDVs has led to its widespread adoption as a covariate in statistical models, assuming a consistent impact across diverse populations^33,34,35^. In fact, SES is typically analyzed and depicted as a monolithic construct, linking low SES with cognitive deprivation and high SES with cognitive enrichment^36,37^. Such broad approaches and their resultant conclusions about social hierarchies imply similar cognitive trajectories for individuals of comparable socioeconomic status, irrespective of nuanced subgroup characteristics, thus ignoring key issues of intersectionality^38^. However, our investigation, by explicit modeling, uncovered and quantified disproportionate SDV effects that render certain subgroups more or less impacted by facets of sociodemographic standing.

Building upon the intersectional theory of cognitive development and stratified populations, Henry et al. discovered that the interplay of race and SES triggers micro-level processes that affect child development^39^. Adding quantitative support to this framework, we contend that the SDV-cognitive development relationship is vaguely defined and inadequately portrays a simple inverse relationship, despite its complexity and strata-specific nature. Disparities were particularly noticeable among Black children, demonstrating that low SES was a strong negative predictor of cognitive development, while high SES exhibited no discriminative nor protective effect^39,40^. Conversely, for White and Asian children, both high and low SES were associated with strong positive effects, challenging the conventional assumption that lower SES universally leads to diminished cognitive responses. This notion aligns with our premise that strata-contexts can directly inform diversity-aware population models.

Racial segregation interacts with structural sociodemographic transformations in society, contributing to the spatial concentration of poverty^14,16,40^. The joint distributions of our model’s state-intercepts, and race-slopes should, in theory, support this premise, as we set out to carefully quantify the link between race and regional stratification in a single, coherent, varying-intercepts and varying-slopes estimation. Our collective findings demonstrate that we can effectively identify spatially concentrated poverty, by pinpointing how our probabilistic model captured key aspects of bias among the examined subgroups. In states with lower predicted cognitive scores, there was a larger population of Black and Hispanic subgroups. Conversely, in states with higher predicted cognitive scores, there was a larger population of White and Asian subgroups. Supporting the premise of spatial inequality theory, we noted that the association between low SES and cognition for Black and Hispanic subgroups residing in predominantly White states was less disadvantageous than in racially-diverse states^41,42,43^. Research indicates that children from low SES backgrounds in counties associated with high upward mobility, defined as the advancement to higher social class, exhibit fewer externalizing behaviors and enhanced cognitive development^44,45,46^. The spatial inequality theory highlights the concept of unequal regional distribution of resources, which might explain why low SES was not a strong negative predictor of cognition in White and Asian families^42,43,47,48^. Our proposed framework illustrates how historical and societal biases are reflected, quantitatively, in population models. Circling back to the foundations of socialization —namely, critical race, social stratification, and spatial inequality theories—our analysis of state-specific outcomes and sociodemographic associations with cognition across race subgroups, helps us understand these social phenomena at the inter-individual level.

A common practice in relevant literature is to draw parallels from trends in Black and White households to juxtapose and characterize how SDVs are intertwined with cognitive development^49^. At the centre of this discourse, the Black-White achievement gap has been cited as a product of race and SES in connection with early childhood development^49,50^. However, our models revealed divergent effects of SDVs on race subgroups, indicating that these effects on cognition are not always directly analogous. The estimated posterior joint distribution of varying-slopes, measuring the race-specific parental marital status effect, revealed that our model’s predictions of cognition were unstable when directly comparing Black and White subgroups. This suggests that parental marital status plays a different role in cognitive development trajectories across race subgroups. Our models effectively quantified the weaker positive effects of high SES on cognitive development in systemically disadvantaged subgroups, a phenomenon known as marginalized-related diminished returns (MDRs)^51,52,53^. Therefore, making the comparison, for example, that children in two-parent households are more likely to be cognitively enriched, than in single-parent households, is not a ground truth that is universally applicable, and may propagate a White-centric narrative onto all race subgroups. These findings accentuate systemic drivers present in society, which our model captures through information from the predictive module of variation in population strata^54,55^. We show that it is distinctly informative to measure the relative effects of SDVs on race and state strata, to elucidate socio-spatial dynamics. In settings where generative models are used for decision-making in healthcare or to guide policy interventions, it’s important to employ a diversity-aware modeling approach to account for intersectional experiences that may be determinant of individual differences within the target population^23,56,57^.

The adoption of generative population models in downstream applications hinges on 4 main aspects: interpretability, reliability, accuracy, and precision^58,59,60^. Although the ABCD Study^©^ was designed to minimize selection bias, it is not guaranteed to be nationally representative across all sociodemographic dimensions^73^. In our diversity-aware modeling framework, the poststratification step is poised for bias mitigation by rebalancing outcomes in view of non-representative population sampling. Poststratification is a statistical technique used to improve the precision and accuracy of estimates by adjusting for known variables within predefined subgroups^24,25,61,62^. Commonly used to reconcile nonresponse bias in survey estimation methods, poststratification serves as a viable mechanism to ultimately enhance the reliability of our analysis, ensuring that the generative predictive samples are representative of our target population^62,63^. We explicitly addressed two primary forms of biases embedded in our model: sampling bias and group attribution bias. The first stems from sampling inefficiencies across nested strata within the ABCD cohort, leading to subgroup representation bias, where race groups in particular states were skewed with respect to their true occurrence in the general population. This caused a domino effect, introducing a second, group attribution bias, encoded within the model itself^64^. This manifested when race and state subgroups were overly hetero- or homogeneous, projecting an SDV profile that perpetuated familiar stereotypes onto those subgroups. Such can be seen in the poststratification results in Pennsylvania, the state that exhibited the most pronounced corrections across all 14 SDV-cognition models. Before poststratification, Pennsylvania, which had the highest percentage of recruited Black participants in the ABCD cohort (52%), was predicted to have lower crystallized, fluid, and overall cognition outcomes than most states. After using the auxiliary US census SDI for de-biasing, Pennsylvania’s predictions were consistent with the average outcomes across all states.

The hierarchical structure of our models was foundational to the effectiveness of the poststratification step, and to deriving insights that were generalizable to underrepresented groups^65,66,67^. By considering both group-level and individual-level variance, these models enabled us to correct our estimates with exceptional granularity. Standalone, Bayesian multilevel regression ensured that over- or underrepresented race and state subgroups in the ABCD sample were drawn closer to their group mean^24,62,63^. When coupled with poststratification, which involved re-weighting our probability estimates based on the known population proportions of each state-race-SDV subgroup, we were able to deduce more accurate conclusions about our target population. For instance, in Missouri, where no Asians were sampled in the ABCD study, the model, after poststratification, provided an estimate into expected sociodemographic effects on cognition for Asians living in Missouri, by pooling and borrowing knowledge from the other data acquisition sites. Consequently, sociodemographic representations were balanced using the US census SDI to ground our model’s predictions in nationally representative estimates. This also contributed to more precise uncertainty intervals of SDV effects on cognitive outcomes across race and state subgroups, contrasting with the biased models where Black and Asian SDV slope effects exhibited wider intervals compared to the parameters for White, Other, and Hispanic subgroups. Therefore, our holistic approach to diversity-aware predictions ensured the identification and mitigation of biases that threatened to compromise our population models. In doing so, our model outputs illuminated socio-spatial effects, reliably interpreting inter-individual differences in cognitive phenotypes, even among the smallest strata in our analysis.

Our investigation into the intricates of cognitive development and sociodemographic factors has yielded profound insights. Through our novel diversity-aware population modeling framework, our analyses revealed the multifaceted interplay between race, geographic location, and SES. By doing so, we’ve circumvented the limitations of conventional strata-agnostic approaches, linking the disproportionate impact of sociodemographic variables on cognitive outcomes across diverse populations. Our quantification of socio-spatial effects within a unified platform has illuminated the marginalized-related diminished returns of high SES among historically underserved subgroups, in line with the theory of social stratification. This underscores the necessity of considering the intrinsic hierarchical structure within populations to capture and derive meaningful insights. Our study represents a paradigm shift in population neuroscience, urging researchers to adopt a more holistic and inclusive approach that encompasses systemic context and the complexity of human diversity.

Our findings underscore the urgent need for future studies to delve deeper into the organizational network of sociodemographic influences on all aspects of population-related research. It is crucial to prioritize intersectionality in our methodologies, recognizing how systemic barriers shape individual experiences in society. A collective effort across research communities to improve the specificity of ethno-racial constructs, as to avoid broad racial categories would bode well for achieving generalizable inter-individual insights. As a proof of principle, leveraging the richly phenotyped ABCD cohort and BMRP to account for population stratification and sample biases, we’ve showcased the reliability, interpretability, accuracy, and precision of our diversity-aware population models -suitable for adoption in downstream applications. This study serves as a catalyst for future research endeavors, propelling us toward a more comprehensive understanding of the sociodemographic determinants of cognitive development and beyond.

### Limitations

The ABCD study, while extensive, employs an oversimplified definition of race and ethnicity, which could impede the generalizability of our findings to underrepresented subsets of the population. Researchers should consider that race is a social construct that continues to evolve through historical and political shifts, as outlined by the Pew Research Center^90^. To anchor analyses in the modern social climate, it is imperative for the scientific community to collaborate on data initiatives to expand the availability of more nuanced and inclusive measurements of race. Acknowledging criticisms of standardized cognitive ability tests for their lack of cultural equivalence^91^ across ethnic groups, we recognize the potential for our diversity-aware population models to learn and perpetuate stereotypes about minorities. Moving forward, concerted efforts addressing the challenges outlined will improve our diversity-aware modeling framework.

## Methods

### ABCD Study

Neurobehavioral and socioeconomic data for this study were sourced from the Adolescent Brain Cognitive Development Study (ABCD; https://abcdstudy.org/) - the largest and most comprehensive biomedical resource on child health and brain development^5^. The ABCD Study is a collaborative aggregation of data from 11,877 children aged 9–10 years (mean age = 9.49 years) across 21 sites in the United States, with participant demographics comprising the following race subgroups: 48% girls, 57% Caucasian, 15% African American, 20% Hispanic, and 8% other ethnicities^68,69^. Baseline measurements were obtained from the ABCD curated 3.0 release, providing comprehensive measures across child and parent domains, including self-reports of race and ethnicity, physical and mental health, neurocognitive performance, sociodemographic factors, cultural values, and environmental conditions^70^. These indicators were characterized using over 6000 deep-profiling assessments, capturing more than 17,000 individual items. (https://data-archive.nimh.nih.gov/abcd). All protocols for the ABCD Study received approval from either a central or site-specific institutional review board committee^70,71^. Caregivers provided written, informed consent, and children offered verbal assent for all research protocols^71^. Additional details about the ABCD Study are available in Garavan et al., 2018. The dataset is funded and managed by the National Institutes of Mental Health Data Archive (NIH) and is openly accessible to qualified researchers. Instructions for data acquisition can be found at https://nda.nih.gov/abcd/request-access.

ABCD employed a multi-stage probability sampling strategy, curating a cohort to closely reflect the sociodemographic composition of the general US population^72^. To minimize systematic sampling and selection biases, a stratified probability sampling method was utilized to ensure randomization and representativeness of selected schools across the US. However, only urban schools were among the 21 collaborating sites, resulting in a relative underrepresentation of rural youth. Despite efforts to create a nationally distributed data resource, the extent to which the ABCD cohort is representative of the US population in terms of race, sex, and SES may vary across different outcome measures^73^.

### Auxiliary Census SDI Data

External census information was obtained from the Robert Graham Center’s Social Deprivation Index (SDI) dataset^74,75^. The SDI quantifies disadvantage in small areas to evaluate associations with health outcomes, and address health inequities (SDI; https://www.graham-center.org/maps-data-tools/social-deprivation-index.html). Butler et al. (2012) developed this index using the 2005–2009 American Community Survey and applied factor analysis of seven demographic characteristics to identify latent factors of social and area deprivation^74,75^. Higher values indicate greater severity, expressed as a composite measure ranging from 0 to 100. Our analysis focused on census tracts among the various geographic areas covered by the SDI, examining 4 of the 7 available demographic characteristics: poverty (less than 100% of the federal poverty line), education level (population percentage with less than 12 years of education), single-parent households, and the percentage of non-employed adults (those not seeking work) under 65 years of age (N=160,000,000 participants). To derive a state-level variable, we mapped census tract codes to their respective states and excluded observations in the SDI from states not included in the ABCD study. To create a race variable, we extracted race percentages (Black, Hispanic, White, Asian, Other) from the most recent US census data for each state. Subsequently, we employed a weighted random choice generator to assign a race attribute to each observation in the SDI, ensuring alignment with real-world race-state distributions.

### Target phenotype and SDV selection

We conducted a cross-sectional analysis of the ABCD study, investigating cognitive development with respect to sociodemographic variables (SDV). To decide which SDV-neurobehavioral relationships to explore, we utilized Pearson’s cross correlation (PCC), charting demographic diversity factors against neurobehavioral phenotypes^76^. Chen et al. illuminated shared brain network features explaining individual variations in childhood behavior^77^. Building upon this work, our PCC encompassed 36 target neurobehavioral phenotypes spanning personality, mental health, and cognitive domains. We then curated a shortlist of 57 sociodemographic diversity factors for inclusion in our PCC, as informed by key variables highlighted by Yip et al. and related ABCD studies, emphasizing their significance in shaping child brain development^78,79^. The resulting PCC was a 36 × 57 matrix, describing the relative linear association between neurobehavioral phenotypes and SDVs. Given correlation coefficients falling within the range of |p| ≤ 0.34, we elected to exclusively model a subset of 14 SDV-behavior pairs meeting our set threshold of |p | ≥ 0.2^80^.

The cognitive domain—fluid, crystallized, and overall cognition phenotypes—exhibited stronger associations with sociodemographic variables (SDVs) compared to phenotypes within the personality and mental health domains. Among the 57 demographic diversity factors charted, education level, poverty occurrence, poverty threshold, parental marital status, and unemployment were the 5 most dominant SDVs. For simplicity, we modeled each of the 14 SDV-cognitive phenotype pairs separately, investigating cognitive variation as a function of the SDV under study in each model. To ensure the robustness of our findings, our inclusion criteria comprised children with complete SDV information and cognitive assessment scores (N=10,900); excluded were participants with missing data or from site 22, as this site was sparsely sampled and discontinued in future ABCD releases. We consolidated 21 sites into 17 states, label encoded the race and state variables, and binarized each SDV based on its respective median value. This procedure was conducted to coherently delineate strata within our sample across state, race, and sociodemographic extremities. Finally, in a two-step process to enhance model interpretability, we applied the natural logarithm to our target cognitive variable to address skewness and performed z-scoring for standardization^81^:

$$Z_{\ln(y)}=\frac{ln\left(y_{0}\right)-\mu_{ln\left(y_{0}\right)}}{s_{ln\left(y_{0}\right)}},$$

where $\mu_{ln\left(y_{0}\right)}$ is the mean of the natural logarithm of our target cognitive variable, and $S_{ln\left(Y_{j}\right)}$ is the standard deviation. Effectively, we systematically quantified cognitive variation relative to the group mean, accounting for major sources of population stratification. Our investigation serves as a proof-of-principle for diversity-aware population modeling, demonstrating that Bayesian multilevel regression and poststratification (BMRP) is a natural candidate for deriving interpretable and reliable insights.

### Bayesian hierarchical model specification

We constructed each of our diversity-aware population models by specifying a varying-intercepts and varying-slopes (VIVS) model to predict continuous cognitive phenotypes. Our BMRP framework extends survey methods commonly used for small-area estimation^24,25,82^. In our VIVS model, we defined 17 state-intercept hierarchies, and 5 race-slope hierarchies. Using Theano shared objects, which allow for data swapping, we instantiated our race and state hierarchies and SDV input variables. Next, we fitted each model on the ABCD sample, then seamlessly incorporated external census SDI data for the poststratification step. Our BMRP approach involved two steps that we carried out for all 14 SDV-cognitive phenotype pairs (cf. previous section), denoted by $y_{i}$ the model outcome, and $x_{i}$, the model input for an individual, i, as described below:

1. Fit a multilevel linear regression model for the individual cognitive prediction $y_{i}$, based on a state-specific average, $\alpha_{j[i]}$, race-specific SDV predictor, $\beta_{j[i]}x_{i}$, and latent individual error, $\sigma^{2}$. In contrast to Gelman et al.’s (2004) logistic regression modeling strategy, in which the authors categorized observations to estimate mean responses for each demographic and state cross-classification, our method retained all observation information to anchor our model in a data-driven way, capturing the full depth of both group- and individual-level variance.
2. From the auxiliary US census SDI dataset, we created our poststratification cells (i.e., consider population strata), c, by aggregating the unique combination of states, races, and the binary SDV predictor, yielding the stratum sample size $\pi_{c}$, for each cell c. We updated our posterior predictive sample using the Theano shared object to swap in the SDI categorized data into our model and obtained a cell probability estimate, $\pi_{c}$, representing the mean prediction for each category in the model. The estimated population average of the cognitive prediction y was thus:
$$\text{by state, s:}\;\theta_{s}=\frac{{\text{Σ}}_{c\epsilon s}\;n_{c}\pi_{c}}{{\text{Σ}}_{c\epsilon s}\;n_{c}}\;\text{by race, r:}\;\theta_{r}=\frac{{\text{Σ}}_{c\epsilon r}\;n_{c}\pi_{c}}{{\text{Σ}}_{c\epsilon r}\;n_{c}},$$
  The state summation was performed over 10 demographic categories (5 race groups × binary SDV, per state), while the race summation involved 34 demographic categories (17 state groups × binary SDV, per race).

### Model Formulation

The cognitive scores $y_{ij}$, for each participant, i, in a race and state strata, j, conform to a normal distribution, characterized by a latent individual mean, $\mu_{ij}$. This mean is expressed as a function of the state-level intercept, $\alpha_{j[i]}$, the race-level slope, $\beta_{j[i]}$, and individual error, $\sigma^{2}$, unexplained by our model. Effectively, to make an individualized prediction, our model estimates: the average cognitive outcome in a participant’s state, the effect of the SDV under study in participants’ race group, and the random model variance:

$$\begin{array}{l} y_{ij}∼N\left(\mu_{ij},\sigma^{2}\right)\\ \mu_{ij}=\alpha_{j[i]}+\beta_{j[i]}\times x_{i}, \end{array}$$

where i represents the index for individual participant observations, j represents the index for group membership, $x_{i}$ denotes the binarized SDV attribute (e.g., education level), $\alpha_{j[i]}$ is the varying-intercepts parameter, estimated for 17 state groups, $\beta_{j[i]}$ and is the slope parameter, estimated for 5 race groups.

### Varying intercepts by state-levels

The group-level intercept $\alpha_{j}$, was partially pooled across 17 states from which the ABCD cohort was sampled, assuming a normal distribution. This varying intercept parameter captured any differences in cognitive scores across different geographical regions, accounting for the shared information among states while allowing for individual state-specific effects on cognitive scores:

$$\alpha_{j}∼N\left(\mu_{\alpha },{\sigma_{\alpha }}^{2}\right),$$

where $\mu_{\alpha }$ estimated the mean of the state-level intercepts, and ${\sigma_{\alpha }}^{2}$ estimated the variance of the state-level intercepts.

### Varying slopes by race-levels

The group-level slope $\beta_{j}$, was partially pooled across 5 race strata in the ABCD study and was assumed to follow a normal distribution. This parameter quantified the relationship between the cognitive phenotype and SDV pair under study for each race subgroup, expressing the confidence and reliability of the estimations. The hierarchical structuring of each binary SDV based on our race strata, enabled contextually dependent predictions:

$$\beta_{j}∼N\left(\mu_{\beta },{\sigma_{\beta }}^{2}\right),$$

where $\mu_{\beta }$ estimated the mean of the race-level slopes, and ${\sigma_{\beta }}^{2}$ estimated the variance of the race-level slopes.

### Weakly Informed Priors

The hyperparameters $\mu_{\alpha }$ and $\mu_{\beta }$ were assumed to be normally distributed, while ${\sigma_{\alpha }}^{2}$ and ${\sigma_{\beta }}^{2}$ were modeled as exponential distributions. These hyperparameters played a crucial role in governing the overall variability and shrinkage of the group-level intercepts and slopes, respectively. This in turn influenced the degree of pooling of information across states and race groups. In the spirit of a data-driven analysis, we assigned weakly informative priors to these hyperparameters^83^. Our hyperpriors, $\tau_{\alpha }$, $\tau_{\beta }$, $\sigma_{\mu \alpha }$, and $\sigma_{\mu \beta }$ expressed the model’s prior beliefs about the variability and central tendencies of the hyperparameters, $\mu_{\alpha }$, $\mu_{\beta }$, ${\sigma_{\alpha }}^{2}$, and ${\sigma_{\beta }}^{2}$. The hyperpriors stroke a balance between allowing the data to influence the model estimation process while incorporating informed expectations:

$$\begin{array}{l} \begin{array}{c} {\mu_{\alpha }}^{∼}N\left(\tau_{\alpha },\sigma_{\mu \alpha }\right)\\ {\sigma_{\alpha }}^{2∼}Exponential\left(\epsilon_{\alpha }\right) \end{array}\\ \begin{array}{c} {\mu_{\beta }}^{∼}N\left(\tau_{\beta },\sigma_{\mu \beta }\right)\\ {\sigma_{\beta }}^{2∼}Exponential\left(\epsilon_{\beta }\right), \end{array} \end{array}$$

where $\tau_{\alpha }$ and $\tau_{\beta }$ denote the mean (equal to zero) of the normal prior distribution and $\sigma_{\mu \alpha }$ and $\sigma_{\mu \beta }$ denote the error (5.0 and 1.0, respectively) of the prior distribution. $\epsilon_{\alpha }$ and $\epsilon_{\beta }$ denote the mean error of the prior error distribution (1.0 and 0.5, respectively).

### MCMC Sampling and Robust Parameter Estimation

Probabilistic hierarchical modeling was specified, implemented, and carried out in the *PyMC3* framework (https://github.com/pymc-devs/pymc3). Joint posterior distributions were approximated using NUTS (No U-Turn Sampler), an efficient Markov Chain Monte Carlo (MCMC) algorithm, following our previous work using Bayesian statistics^84,85,86^. MCMC methods enabled robust parameter estimation, allowing the model to draw accurate posterior distributions for nuanced and reliable inferences.

NUTS employs an adaptive approach to determine the path of the Markov chain during sampling. This sampler efficiently explores high-dimensional parameter spaces by dynamically adjusting the trajectory, eliminating the need for manual tuning of parameters (e.g., step size). This results in faster convergence and more accurate approximations of joint posterior distributions, making this solution advantageous for complex modeling scenarios. We drew 2,000 samples from the joint posterior distribution over all parameters in the model, refining the estimations at each step to converge toward the desired target distribution. Depending on model convergence, tuning ranged from 2,000 to 3,000 steps. At every stage of the MCMC chain, a comprehensive estimation of the entire set of parameter values was carried out, sharpening their joint credibility with respect to the observed data. We confirmed model convergence by examining overlap in the geometry of posterior parameter distributions from four independent MCMC chains. Finally, we plotted our resulting Bayesian posterior parameter distributions using *Arviz Python package* (https://www.arviz.org/).

### Model Evaluation and Predictive Power

Our model quality was evaluated by inspecting the i) $\hat{R}$ quality criteria and ii) effective sample size. We further scrutinized how well the model captured the intricate complexities in observed cognitive outcomes by iii) performing posterior predictive checks (PPC). Simulating new data based on the estimated parameter posteriors, our PPCs provided a comprehensive evaluation of the model’s predictive performance, allowing us to assess its ability to replicate the patterns observed in the actual data^87,88^. We used the Bayesian R^2^ score from Arviz to compare the variance of the observed data to the posterior predictive distribution variance^30^. This served as confirmation that the posterior predictive distribution of the model was well-fitted to the observed data, which is essential as a precondition for domain interpretability of the obtained model. To validate the poststratified model, we conducted sensitivity analyses to assess the impact of different poststratification scenarios on the robustness of our predictions. This comprehensive strategy enhances the model’s utility across diverse demographic contexts, allowing for more nuanced and accurate predictions of cognitive outcomes.

### Poststratification Strategy

The poststratification strategy was a crucial methodological step to enhance the precision and accuracy of our model’s predictions^24,25,82^. We aimed to rectify potential biases related to group attribution, such as systematic skewing in recruiting specific population strata in the ABCD cohort (e.g., over- or under-representation of demographically defined subgroups). Thus, poststratification was employed to ensure that the final posterior predictive sample was representative of the entire population, and equally reliable for smaller subpopulations. For this technique, we leveraged auxiliary US census SDI data (cf. above), then constructed a poststratification matrix by dividing the population into cells based on subgroup membership. An example race-state-SDV category would be a white, Californian, raised by married parents, for which we know the population proportion of individuals in that category. The cell would act as a placeholder for the mean prediction of that category and the known US census SDI stratum weight to be applied.

Poststratification, as the name suggests, was implemented after model fitting (cf. above), using the set of joint posterior parameter estimates as the basis for the final analysis. Each of our 14 models had 170 cells corresponding to the unique combination of 17 states, 5 races, and the binary SDV under study. To fill the poststratification matrix, we retrieved the probability estimate $\pi_{c}$, which is the mean model prediction of y, for a particular category stratum. Given the estimated joint posterior parameter distributions that make up our model (cf. above), we obtained $\pi_{c}$ by re-instantiating our race and state hierarchies and SDV input variables. The Theano shared objects facilitated dynamic swapping of the ABCD data and US census SDI, enabling us to re-run our posterior predictions. From this drew 8000 samples for each category, yielding an 8000 × 170 poststratification matrix.

Next, the stratum weights were applied to the cell probability estimates using the known category population proportions derived from the auxiliary US census SDI. We calculated the data weighted average across 10 and 34 categories for each race- and state-specific poststratification (cf. above), respectively. This meant that the adjusted estimates for a target population were slightly different when the data-weighted average focused on either the state or race summary. Overall, poststratification ensured that our model was not only accurate and precise, but also sensitive to the diversity present in different demographic subgroups. The unique quality of the Bayesian paradigm being able to shrink group and individual effects towards a common shared effect to varying degrees ensured that over or underrepresented cells were properly accounted for in our framework.

This post-stratification scheme was repeated for all 14 SDV-behavior pairs (cf. above). Our comprehensive strategy enhanced the model’s utility across diverse demographic contexts, refining our model parameters and predictions to reflect the distribution of sociodemographic characteristics in the target population.

## Data Availability

The data supporting the findings of this study are available from the Adolescent Brain Cognitive Development (ABCD) dataset. The ABCD dataset is a publicly available resource accessible through the National Institute of Mental Health Data Archive (NDA). All relevant instructions to obtain the data can be found online (https://nda.nih.gov/abcd/request-access). The specific data used in this study can be located within the ABCD dataset under X.
